# 228. Epidemiology and Susceptibility Profiles of ST131-O25b *Escherichia coli* Detected Among Cephalosporin and/or Carbapenem-Resistant Isolates Collected in United States Hospitals

**DOI:** 10.1093/ofid/ofab466.430

**Published:** 2021-12-04

**Authors:** Mariana Castanheira, Mariana Castanheira, Timothy B Doyle, Andrew P Davis, Valerie Kantro, Christopher M Rubino, Sujata M Bhavnani, Paul G Ambrose

**Affiliations:** 1 JMI Laboratories, North Liberty, IA; 2 Institute for Clinical Pharmacodynamics, Inc., Schenectady, NY

## Abstract

**Background:**

ST131 *Escherichia coli* possess virulence genes, adaptability for human colonization and are often associated to resistance genes. We evaluated the prevalence, O-antigen and susceptibility profiles of ST131 among β-lactam resistant *E. coli* isolates collected in US hospitals.

**Methods:**

A total of 6,768 *E. coli* isolates collected during 2017 and 2018 were susceptibility tested using reference broth microdilution method. Among these, 1,154 displayed MIC values >1 mg/L against 2 of the following: ceftazidime, ceftriaxone, cefepime or aztreonam or resistance to imipenem or meropenem (CLSI breakpoints) and were submitted to whole genome sequencing (WGS) and data analysis (MLST and O antigen). The genes *wzx*, *wzy*, *wzm*, and *wzt* were used to identify the O-antigen. Reference guided assembly for gene cluster O-AGC was used to differentiate O25a and O25b.

**Results:**

Among the WGS *E. coli* isolates, 627 (54.3%) belonged to ST131 or were single loci variants (SLVs; 1 allele difference). A total of 586 (93.5% of ST131 and 50.8% of sequenced isolates) belonged to the O25b serotype. The remaining 41 isolates belonged to serotypes O16 (40 isolates) or O153var1 (1). ST131 isolates and O25b isolates were considerably more resistant to fluoroquinolones (92.0%-93.3% and 94.7%-95.5%, respectively) when compared to the overall WGS isolate collection (75.6%-77.0%). ST131-O25b isolates were more resistant to 12 /16 antimicrobial agents analyzed, including all β-lactam agents (0.9%-12.7% more resistant), fluoroquinolones (34.5-41.0%), and aminoglycosides (1.2-37.3%). ST131 (49.0%) and ST131-O25b (51.5%) isolates had higher multi-drug resistant (MDR) rates compared to all *E. coli* isolates (7.3%), WGS isolates (41.4%), and isolates that did not carry these traits (32.4% for non-ST131 and 12.2% for non-O25b).

**Conclusion:**

ST131 and ST131-O25b *E. coli* isolates were common among β-lactam resistant *E. coli* from US hospitals. These isolates were significantly more resistant than their counterparts, despite the elevated resistance rates of the overall WGS collection. ST131-O25b *E. coli* isolates have the potential to present a challenge for antimicrobial treatment. Specific therapies that are effective against these isolates should be investigated.

**Disclosures:**

**Mariana Castanheira, PhD**, **AbbVie (formerly Allergan**) (Research Grant or Support)**Bravos Biosciences** (Research Grant or Support)**Cidara Therapeutics, Inc.** (Research Grant or Support)**Cipla Therapeutics** (Research Grant or Support)**Cipla USA Inc.** (Research Grant or Support)**GlaxoSmithKline** (Research Grant or Support)**Melinta Therapeutics, Inc.** (Research Grant or Support)**Melinta Therapeutics, LLC** (Research Grant or Support)**Pfizer, Inc.** (Research Grant or Support)**Qpex Biopharma** (Research Grant or Support)**Shionogi** (Research Grant or Support)**Spero Therapeutics** (Research Grant or Support) **Mariana Castanheira, PhD**, Affinity Biosensors (Individual(s) Involved: Self): Research Grant or Support; Allergan (Individual(s) Involved: Self): Research Grant or Support; Amicrobe, Inc (Individual(s) Involved: Self): Research Grant or Support; Amplyx Pharma (Individual(s) Involved: Self): Research Grant or Support; Artugen Therapeutics USA, Inc. (Individual(s) Involved: Self): Research Grant or Support; Astellas (Individual(s) Involved: Self): Research Grant or Support; Basilea (Individual(s) Involved: Self): Research Grant or Support; Beth Israel Deaconess Medical Center (Individual(s) Involved: Self): Research Grant or Support; BIDMC (Individual(s) Involved: Self): Research Grant or Support; bioMerieux Inc. (Individual(s) Involved: Self): Research Grant or Support; BioVersys Ag (Individual(s) Involved: Self): Research Grant or Support; Bugworks (Individual(s) Involved: Self): Research Grant or Support; Cidara (Individual(s) Involved: Self): Research Grant or Support; Cipla (Individual(s) Involved: Self): Research Grant or Support; Contrafect (Individual(s) Involved: Self): Research Grant or Support; Cormedix (Individual(s) Involved: Self): Research Grant or Support; Crestone, Inc. (Individual(s) Involved: Self): Research Grant or Support; Curza (Individual(s) Involved: Self): Research Grant or Support; CXC7 (Individual(s) Involved: Self): Research Grant or Support; Entasis (Individual(s) Involved: Self): Research Grant or Support; Fedora Pharmaceutical (Individual(s) Involved: Self): Research Grant or Support; Fimbrion Therapeutics (Individual(s) Involved: Self): Research Grant or Support; Fox Chase (Individual(s) Involved: Self): Research Grant or Support; GlaxoSmithKline (Individual(s) Involved: Self): Research Grant or Support; Guardian Therapeutics (Individual(s) Involved: Self): Research Grant or Support; Hardy Diagnostics (Individual(s) Involved: Self): Research Grant or Support; IHMA (Individual(s) Involved: Self): Research Grant or Support; Janssen Research & Development (Individual(s) Involved: Self): Research Grant or Support; Johnson & Johnson (Individual(s) Involved: Self): Research Grant or Support; Kaleido Biosceinces (Individual(s) Involved: Self): Research Grant or Support; KBP Biosciences (Individual(s) Involved: Self): Research Grant or Support; Luminex (Individual(s) Involved: Self): Research Grant or Support; Matrivax (Individual(s) Involved: Self): Research Grant or Support; Mayo Clinic (Individual(s) Involved: Self): Research Grant or Support; Medpace (Individual(s) Involved: Self): Research Grant or Support; Meiji Seika Pharma Co., Ltd. (Individual(s) Involved: Self): Research Grant or Support; Melinta (Individual(s) Involved: Self): Research Grant or Support; Menarini (Individual(s) Involved: Self): Research Grant or Support; Merck (Individual(s) Involved: Self): Research Grant or Support; Meridian Bioscience Inc. (Individual(s) Involved: Self): Research Grant or Support; Micromyx (Individual(s) Involved: Self): Research Grant or Support; MicuRx (Individual(s) Involved: Self): Research Grant or Support; N8 Medical (Individual(s) Involved: Self): Research Grant or Support; Nabriva (Individual(s) Involved: Self): Research Grant or Support; National Institutes of Health (Individual(s) Involved: Self): Research Grant or Support; National University of Singapore (Individual(s) Involved: Self): Research Grant or Support; North Bristol NHS Trust (Individual(s) Involved: Self): Research Grant or Support; Novome Biotechnologies (Individual(s) Involved: Self): Research Grant or Support; Paratek (Individual(s) Involved: Self): Research Grant or Support; Pfizer (Individual(s) Involved: Self): Research Grant or Support; Prokaryotics Inc. (Individual(s) Involved: Self): Research Grant or Support; QPEX Biopharma (Individual(s) Involved: Self): Research Grant or Support; Rhode Island Hospital (Individual(s) Involved: Self): Research Grant or Support; RIHML (Individual(s) Involved: Self): Research Grant or Support; Roche (Individual(s) Involved: Self): Research Grant or Support; Roivant (Individual(s) Involved: Self): Research Grant or Support; Salvat (Individual(s) Involved: Self): Research Grant or Support; Scynexis (Individual(s) Involved: Self): Research Grant or Support; SeLux Diagnostics (Individual(s) Involved: Self): Research Grant or Support; Shionogi (Individual(s) Involved: Self): Research Grant or Support; Specific Diagnostics (Individual(s) Involved: Self): Research Grant or Support; Spero (Individual(s) Involved: Self): Research Grant or Support; SuperTrans Medical LT (Individual(s) Involved: Self): Research Grant or Support; T2 Biosystems (Individual(s) Involved: Self): Research Grant or Support; The University of Queensland (Individual(s) Involved: Self): Research Grant or Support; Thermo Fisher Scientific (Individual(s) Involved: Self): Research Grant or Support; Tufts Medical Center (Individual(s) Involved: Self): Research Grant or Support; Universite de Sherbrooke (Individual(s) Involved: Self): Research Grant or Support; University of Iowa (Individual(s) Involved: Self): Research Grant or Support; University of Iowa Hospitals and Clinics (Individual(s) Involved: Self): Research Grant or Support; University of Wisconsin (Individual(s) Involved: Self): Research Grant or Support; UNT System College of Pharmacy (Individual(s) Involved: Self): Research Grant or Support; URMC (Individual(s) Involved: Self): Research Grant or Support; UT Southwestern (Individual(s) Involved: Self): Research Grant or Support; VenatoRx (Individual(s) Involved: Self): Research Grant or Support; Viosera Therapeutics (Individual(s) Involved: Self): Research Grant or Support; Wayne State University (Individual(s) Involved: Self): Research Grant or Support **Timothy B. Doyle**, **AbbVie (formerly Allergan**) (Research Grant or Support)**Bravos Biosciences** (Research Grant or Support)**GlaxoSmithKline** (Research Grant or Support)**Melinta Therapeutics, Inc.** (Research Grant or Support)**Pfizer, Inc.** (Research Grant or Support)**Shionogi** (Research Grant or Support)**Spero Therapeutics** (Research Grant or Support) **Andrew P. Davis, BS**, **Bravos Biosciences** (Research Grant or Support) **Valerie Kantro, BS**, **Bravos Biosciences** (Research Grant or Support) **Christopher M. Rubino, Pharm.D.**, **3-V Biosciences** (Grant/Research Support)**Achogen** (Grant/Research Support)**Amplyx Pharmaceuticals, Inc.** (Grant/Research Support)**Arixa Pharmaceuticals** (Grant/Research Support)**Arsanis Inc.** (Grant/Research Support)**B. Braun Medical Inc.** (Grant/Research Support)**Basilea Pharmaceutica** (Grant/Research Support)**BLC USA** (Research Grant or Support)**Boston Pharmaceuticals** (Grant/Research Support)**Bravos Biosciences, LLC** (Grant/Research Support, Other Financial or Material Support, member/owner)**Cidara Therapeutics Inc.** (Grant/Research Support)**Cipla, USA** (Grant/Research Support)**Corcept Therapeutics** (Grant/Research Support)**Cumberland Pharmaceuticals** (Grant/Research Support)**Debiopharm International SA** (Grant/Research Support)**Discuva Limited** (Grant/Research Support)**Emerald Lake Technologies** (Grant/Research Support)**Enhanced Pharmacodynamics** (Grant/Research Support)**Entasis Therapeutics** (Grant/Research Support)**E-Scape Bio** (Grant/Research Support)**Genentech** (Grant/Research Support)**Geom Therapeutics, Inc.** (Grant/Research Support)**GlaxoSmithKline** (Grant/Research Support)**Hoffmann-La Roche** (Grant/Research Support)**Horizon Orphan LLC** (Grant/Research Support)**ICPD Biosciences, LLC** (Grant/Research Support, Other Financial or Material Support, member/owner)**Indalo Therapeutics** (Grant/Research Support)**Insmed Inc.** (Grant/Research Support)**Institute for Clinical Pharmacodynamics** (Employee)**Iterum** (Grant/Research Support)**KBP Biosciences USA** (Grant/Research Support)**Kyoto Biopharma, Inc.** (Grant/Research Support)**Matinas** (Grant/Research Support)**Meiji Seika Pharma Co., Ltd.** (Grant/Research Support)**Melinta Therapeutics** (Grant/Research Support)**Menarini Ricerche S.p.A.** (Grant/Research Support)**Merck & Co., Inc** (Grant/Research Support)**Mutabilis** (Grant/Research Support)**Nabriva Therapeutics AG** (Grant/Research Support)**Naeja-RGM Pharmaceuticals** (Grant/Research Support)**Nosopharm SAS** (Grant/Research Support)**Novartis Pharmaceuticals Corp.** (Grant/Research Support)**NuCana Biomed** (Grant/Research Support)**Paratek Pharmaceuticals, Inc.** (Grant/Research Support)**Polyphor, Ltd.** (Grant/Research Support)**Prothena Corporation** (Grant/Research Support)**PTC Therapeutics** (Grant/Research Support)**Rempex Pharmaceuticals** (Grant/Research Support)**Roche TCRC** (Grant/Research Support)**Sagimet** (Grant/Research Support)**scPharmaceuticals Inc.** (Grant/Research Support)**Scynexis** (Grant/Research Support)**Spero Therapeutics** (Grant/Research Support)**TauRx Therapeutics** (Grant/Research Support)**Tetraphase Pharmaceuticals** (Grant/Research Support)**Theravance Biopharma Pharmaceutica** (Grant/Research Support)**USCAST** (Grant/Research Support)**VenatoRx** (Grant/Research Support)**Vical Incorporated** (Grant/Research Support)**Wockhardt Bio AG** (Grant/Research Support)**Zavante Therapeutics** (Grant/Research Support)**Zogenix International** (Grant/Research Support) **Sujata M. Bhavnani, Pharm.D., M.S., FIDSA**, **3-V Biosciences** (Grant/Research Support)**Achogen** (Grant/Research Support)**Amplyx Pharmaceuticals, Inc.** (Grant/Research Support)**Arixa Pharmaceuticals** (Grant/Research Support)**Arsanis Inc.** (Grant/Research Support)**B. Braun Medical Inc.** (Grant/Research Support)**Basilea Pharmaceutica** (Grant/Research Support)**BLC USA** (Grant/Research Support)**Boston Pharmaceuticals** (Grant/Research Support)**Bravos Biosciences, LLC** (Grant/Research Support, Other Financial or Material Support, member/owner)**Cidara Therapeutics Inc.** (Grant/Research Support)**Cipla, USA** (Grant/Research Support)**Corcept Therapeutics** (Grant/Research Support)**Cumberland Pharmaceuticals** (Grant/Research Support)**Debiopharm International SA** (Grant/Research Support)**Discuva Limited** (Grant/Research Support)**Emerald Lake Technologies** (Grant/Research Support)**Enhanced Pharmacodynamics** (Grant/Research Support)**Entasis Therapeutics** (Grant/Research Support)**E-Scape Bio** (Grant/Research Support)**Genentech** (Grant/Research Support)**Geom Therapeutics, Inc.** (Grant/Research Support)**GlaxoSmithKline** (Grant/Research Support)**Hoffmann-La Roche** (Grant/Research Support)**Horizon Orphan LLC** (Grant/Research Support)**ICPD Biosciences, LLC** (Grant/Research Support, Other Financial or Material Support, member/owner)**Indalo Therapeutics** (Grant/Research Support)**Insmed Inc.** (Grant/Research Support)**Institute for Clinical Pharmacodynamics** (Employee)**Iterum** (Grant/Research Support)**KBP Biosciences USA** (Grant/Research Support)**Kyoto Biopharma, Inc.** (Grant/Research Support)**Matinas** (Grant/Research Support)**Meiji Seika Pharma Co., Ltd.** (Grant/Research Support)**Melinta Therapeutics** (Grant/Research Support)**Menarini Ricerche S.p.A.** (Grant/Research Support)**Merck & Co., Inc** (Grant/Research Support)**Mutabilis** (Grant/Research Support)**Nabriva Therapeutics AG** (Grant/Research Support)**Naeja-RGM Pharmaceuticals** (Grant/Research Support)**Nosopharm SAS** (Grant/Research Support)**Novartis Pharmaceuticals Corp.** (Grant/Research Support)**NuCana Biomed** (Grant/Research Support)**Paratek Pharmaceuticals, Inc.** (Grant/Research Support)**Polyphor, Ltd.** (Grant/Research Support)**Prothena Corporation** (Grant/Research Support)**PTC Therapeutics** (Grant/Research Support)**Rempex Pharmaceuticals** (Grant/Research Support)**Roche TCRC** (Grant/Research Support)**Sagimet** (Grant/Research Support)**scPharmaceuticals Inc.** (Grant/Research Support)**Scynexis** (Grant/Research Support)**Spero Therapeutics** (Grant/Research Support)**TauRx Therapeutics** (Grant/Research Support)**Tetraphase Pharmaceuticals** (Grant/Research Support)**Theravance Biopharma Pharmaceutica** (Grant/Research Support)**USCAST** (Grant/Research Support)**VenatoRx** (Grant/Research Support)**Vical Incorporated** (Grant/Research Support)**Wockhardt Bio AG** (Grant/Research Support)**Zavante Therapeutics** (Grant/Research Support)**Zogenix International** (Grant/Research Support) **Paul G. Ambrose, Pharm.D., FIDSA**, **3-V Biosciences** (Grant/Research Support)**Achogen** (Grant/Research Support)**Amplyx Pharmaceuticals, Inc.** (Grant/Research Support)**Arixa Pharmaceuticals** (Grant/Research Support)**Arsanis Inc.** (Grant/Research Support)**B. Braun Medical Inc.** (Grant/Research Support)**Basilea Pharmaceutica** (Grant/Research Support)**BLC USA** (Grant/Research Support)**Boston Pharmaceuticals** (Grant/Research Support)**Bravos Biosciences, LLC** (Grant/Research Support, Other Financial or Material Support, member/owner)**Cidara Therapeutics Inc.** (Grant/Research Support)**Cipla, USA** (Grant/Research Support)**Corcept Therapeutics** (Grant/Research Support)**Cumberland Pharmaceuticals** (Grant/Research Support)**Debiopharm International SA** (Grant/Research Support)**Discuva Limited** (Research Grant or Support)**Emerald Lake Technologies** (Grant/Research Support)**Enhanced Pharmacodynamics** (Grant/Research Support)**Entasis Therapeutics** (Grant/Research Support)**E-Scape Bio** (Grant/Research Support)**Genentech** (Grant/Research Support)**Geom Therapeutics, Inc.** (Grant/Research Support)**GlaxoSmithKline** (Grant/Research Support)**Hoffmann-La Roche** (Grant/Research Support)**Horizon Orphan LLC** (Grant/Research Support)**ICPD Biosciences, LLC** (Grant/Research Support, Other Financial or Material Support, member/owner)**Indalo Therapeutics** (Grant/Research Support)**Insmed Inc.** (Grant/Research Support)**Institute for Clinical Pharmacodynamics** (Employee)**Iterum** (Grant/Research Support)**KBP Biosciences USA** (Grant/Research Support)**Kyoto Biopharma, Inc.** (Grant/Research Support)**Matinas** (Grant/Research Support)**Meiji Seika Pharma Co., Ltd.** (Grant/Research Support)**Melinta Therapeutics** (Grant/Research Support)**Menarini Ricerche S.p.A.** (Grant/Research Support)**Merck & Co., Inc** (Grant/Research Support)**Mutabilis** (Grant/Research Support)**Nabriva Therapeutics AG** (Grant/Research Support)**Naeja-RGM Pharmaceuticals** (Grant/Research Support)**Nosopharm SAS** (Grant/Research Support)**Novartis Pharmaceuticals Corp.** (Grant/Research Support)**NuCana Biomed** (Grant/Research Support)**Paratek Pharmaceuticals, Inc.** (Grant/Research Support)**Polyphor, Ltd.** (Grant/Research Support)**Prothena Corporation** (Grant/Research Support)**PTC Therapeutics** (Grant/Research Support)**Rempex Pharmaceuticals** (Grant/Research Support)**Roche TCRC** (Grant/Research Support)**Sagimet** (Grant/Research Support)**scPharmaceuticals Inc.** (Grant/Research Support)**Scynexis** (Grant/Research Support)**Spero Therapeutics** (Grant/Research Support)**TauRx Therapeutics** (Grant/Research Support)**Tetraphase Pharmaceuticals** (Grant/Research Support)**Theravance Biopharma Pharmaceutica** (Grant/Research Support)**USCAST** (Grant/Research Support)**VenatoRx** (Grant/Research Support)**Vical Incorporated** (Grant/Research Support)**Wockhardt Bio AG** (Grant/Research Support)**Zavante Therapeutics** (Grant/Research Support)**Zogenix International** (Grant/Research Support)

